# Simultaneous Determination of Cyclosporine A, Tacrolimus, Sirolimus, and Everolimus in Whole-Blood Samples by LC-MS/MS

**DOI:** 10.1100/2012/571201

**Published:** 2012-05-02

**Authors:** Mustafa Karapirli, Murat Kizilgun, Ozgur Yesilyurt, Husamettin Gul, Zeki Ilker Kunak, Emin Ozgur Akgul, Enis Macit, Tuncer Cayci, Yasemin Gulcan Kurt, Ibrahim Aydin, Hakan Yaren, Melik Seyrek, Erdinc Cakir, Halil Yaman

**Affiliations:** ^1^Council of Forensic Medicine, Ankara Branch, Kecioren, 06018 Ankara, Turkey; ^2^Department of Biochemistry, Diskapi Children' Health and Diseases, Hematology, Oncology Training and Research Hospital, Ministry of Health, 06590 Ankara, Turkey; ^3^Department of Pharmacology, Gulhane Military Medical Academy, 06018 Ankara, Turkey; ^4^Department of Medical Chemical/Biological/Radiological/Nuclear Defense, Gulhane Military Medical Academy, Etlik, 06018 Ankara, Turkey; ^5^Department of Clinical Biochemistry, Gulhane Military Medical Academy, 06018 Ankara, Turkey; ^6^Department of Clinical Chemistry, Gulhane Military Medical Academy, Etlik, 06018 Ankara, Turkey

## Abstract

*Objectives*. Cyclosporine A (CyA), tacrolimus (TRL), sirolimus (SIR), and everolimus (RAD) are immunosuppressive drugs frequently used in organ transplantation. Our aim was to confirm a robust sensitive and selective liquid chromatography-tandem mass spectrometry (LC-MS/MS) method for determination of CyA, TRL, SIR, and RAD in whole-blood samples. *Materials and Methods*. We used an integrated online solid-phase extraction-LC-MS/MS system and atmospheric pressure ionization tandem mass spectrometry (API-MS/MS) in the multiple reaction monitoring (MRM) detection mode. CyA, TRL, SIR, and RAD were simultaneously analyzed in whole blood treated with precipitation reagent taken from transplant patients. *Results*. System performance parameters were suitable for using this method as a high-throughput technique in clinical practice. The high concentration of one analyte in the sample did not affect the concentration of other analytes. Total analytical time was 2.5 min, and retention times of all analytes were shorter than 2 minutes. *Conclusion*. This LC-MS/MS method can be preferable for therapeutic drug monitoring of these immunosuppressive drugs (CyA, TRL, SRL, and RAD) in whole blood. Sample preparation was too short and simple in this method, and it permits robust, rapid, sensitive, selective, and simultaneous determination of these drugs.

## 1. Introduction

Cyclosporine A (CyA), tacrolimus (TRL), sirolimus (SRL), and everolimus (RAD) are the most frequently used immunosuppressive drugs in organ transplantation [1]. CyA and TRL act as calcineurin inhibitors and cause lymphocyte proliferation downstream via cytokine production suppressing [2]. SRL and RAD inhibit T-cell cycle progression by blocking interleukin-2 production [3]. These immunosuppressive drugs have narrow therapeutic ranges. They may cause numerous side effects including immunological, renal, hepatic, and neurological complications, requiring dose adjustment or discontinuation in a significant percentage of patients [4, 5]. In addition, there is important variation for blood levels of these immunosuppressive drugs in different individuals, and ethnicities may also affect these parameters [6, 7]. Therefore, therapeutic drug monitoring (TDM) plays a key role in maintaining therapeutic blood and plasma levels of immunosuppressive drugs, which has narrow therapeutic ranges for reducing their risk of toxicity and organ rejection [8]. TDM has been used to monitor drug levels in routine patient care. The methodology of TDM must be precise and accurate for immunosuppressive drugs [9]. Immunosuppressive drugs have some complementary mechanisms of action and interact synergistically when used together; therefore they are often combined in clinical practice [4]. Due to increasing number of transplant patients and of combined drugs used, simultaneous determination of these drugs is required for routine TDM.

There are two main analytical methods for determination of immunosuppressive drugs in transplant patients: immunoassays (microparticle enzyme immunoassay, cloned enzyme donor immunoassay, etc.) and liquid chromatography-based methods (high-performance liquid chromatography (HPLC) with ultraviolet detection, LC-mass spectrometry (LC-MS), and LC-tandem mass spectrometry (LC-MS/MS)) [10]. Traditionally most routine laboratories use immunological methods for quantification of immunosuppressive drugs. Due to cross-reactions with some metabolites of these drugs, overestimation of the concentrations is a major problem in immunological techniques [9, 11]. LC-MS/MS has some advantages compared to other methods, and its cost has also been decreased, recently. This method is more specific and sensitive than immunological methods for these immunosuppressive drugs. Simultaneous measurements of several drugs can also be possible with LC-MS/MS technique [7]. Because of the above mentioned reasons, LC-MS/MS is generally accepted as the technique of choice [12]. Various LC-MS and LC-MS/MS methods have been developed and used for TDM in daily clinical routine laboratories [11–23].

The aim of this study was to confirm a rapid, sensitive, selective, and cost-effective LC-MS/MS method, which is able to measure CyA, TRL, SRL, and RAD in whole-blood samples for the purpose of TDM.

## 2. Methods

The method described by Koal et al. was established in our laboratory for determination of CyA, TRL, SRL, and RAD in this study [14]. EDTA-treated excess blood specimens of organ transplant patients (kidney and bone marrow) were analyzed immediately in the same day at the LC-MS/MS laboratory. All analyses performed within 6 hours of venous blood drawing.

### 2.1. Chemicals

HPLC grade methanol, ammonium acetate, and zinc sulfate heptahydrate were purchased from Merck (Darmstadt, Germany), and acetic acid was provided from Riedel-de Haen (Hannover-Seelze, Germany). Ultrapure water was supplied by a Milli-Q Water Purification System from Millipore (Molsheim, France). Ascomycin, SRL, RAD, TRL, Cyclosporine D (CyD), and CyA were obtained from ImmuChrom (Heppenheim, Germany). All reagents and solvents were of analytical grade.

Commercially available whole-blood calibrators (6 multilevel calibrator set) and quality control (QC) samples (three-level whole blood controls) were purchased from Immuchrom (Heppenheim, Germany). They were used for calibration of CyA, SRL, TRL, and RAD assays. Concentration ranges were between 0 and 39.70 ng/mL (0.0, 1.22, 2.46, 5.03, 10.20, and 39.70) for TRL, between 0 and 44.30 ng/mL (0.0, 1.38, 2.71, 5.41, 11.30, and 44.30) for SRL, between 0 and 44.90 ng/mL (0.0, 1.20, 2.62, 5.13, 10.90, and 44.90) for RAD, and between 0 and 1345 ng/mL (0.0. 24.30, 48.40, 92.40, 187.0, and 1345.0) for CyA. All calibrators and quality control samples were aliquoted in 100 *μ*L portions in eppendorf tubes and immediately stored at −80°C until analysis. Batches were stable for at least 90 days. One hour before sample preparation, one batch of calibrators and quality control samples were thawed. The usage of commercial calibrators eliminates an important source of random errors, compared to the preparation of “in house” standards.

### 2.2. Sample Preparation

Precipitation reagent including internal standards (ascomycin and CyD) was prepared immediately before sample preparation. Precipitation reagent used in this study was methanol/1.125 M ZnSO_4_ in water (66/34, v/v) including 20 ng/mL Ascomycin and 125 ng/mL CyD. All specimens (EDTA-treated whole blood samples, calibrators, and controls) were put into a 1.5 mL conical test tube as 100 *μ*L volume and 200 *μ*L precipitation reagent was added. Samples were immediately vortexed 30 sec and left 5 min at the room temperature. After vortexing for additional 5 sec, the tubes were centrifuged for 10 min at 15000 ×g at 4°C. Supernatants were transferred into the autosampler vials. The temperature of autosampler was adjusted at 20°C during the analysis for providing standard experimental conditions.

### 2.3. Instrumentation

**Figure 1:**
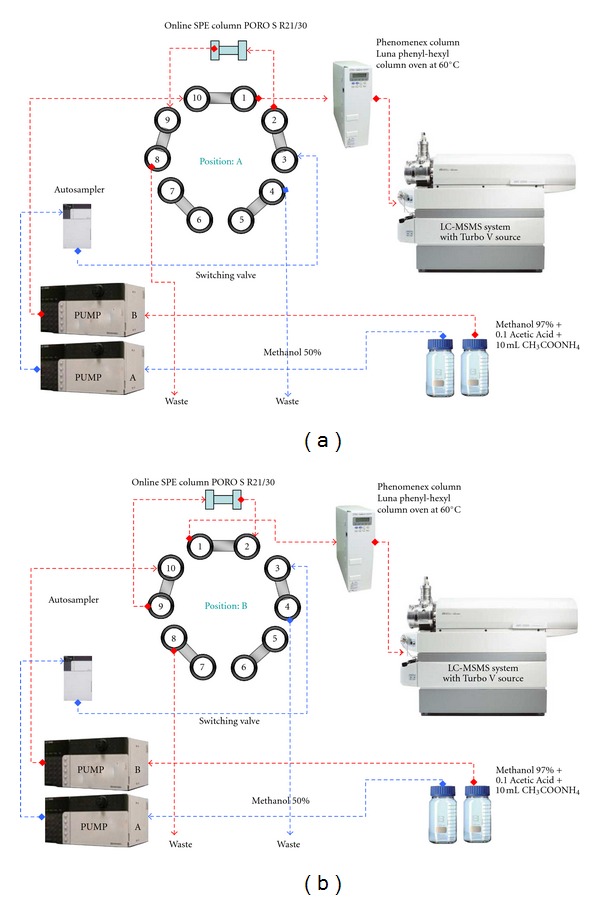
Column switching procedure of the online SPE-LC-MS/MS setup: (a) sample loading and clean-up; (b) sample elution from the analytical column.

A Shimadzu Prominence series Ultra Fast Liquid Chromatography (UFLC) system (Kyoto, Japan) equipped with a temperature controlled autosampler (Shimadzu Prominence Series SIL 20AC HT, autosampler) was used. Two isocratic UFLC pumps (Shimadzu Prominence 20 AD series pump, Kyoto, Japan) were used. HPLC separation was performed with column switching system and two isocratic UFLC pumps (Shimadzu Prominence 20 AD series pump, Kyoto, Japan). 10-port valve (Valco) controlled by a software were used for column switching, but only seven of these ports were used (Figure 1).

### 2.4. LC-MS/MS

It has been shown that combination of LC and tandem mass spectrometry has advantage of simultaneous measurement of different immunosuppressive drugs [16]. But use of these methods in routine TDM has some disadvantages, for example, extraction of drugs from blood requires long time. For the sake of robust and rapid cleaning procedure, automatic online solid phase extraction was used in this study.

For online sample clean-up, a perfusion column (POROS R1/20, 2.1 × 30 mm, 20 *μ*m particle size, Applied Biosystems, Darmstadt, Germany) was used. The use of this column provides high rate of washing, flushing, and re-equilibration, so that there was no waste of time between SPE and chromatographic separation. Phenyl Hexyl RP column (Phenomenex Luna 5 *μ*m particle size, 2 × 50 mm, Aschaffenburg, Germany) was used as an analytical column. HPLC separation was performed at 60°C with an HPLC-column oven (Shimadzu Prominence, CTO 10 AS vp Column oven). A triple quadrupole mass spectrometer (API 3200 Applied Biosystems/MDS Sciex Concord, Canada) with TurboIonSpray source (ESI) was used in positive ion mode. Multiple reaction mode (MRM) was performed for all specimens.

Loading and injecting diagrams for the samples on the switching valve for online SPE column are shown in Figure 1. Two mobile phases, eluent A (MeOH/H_2_O (50/50, v/v)) and eluent B (high organic solvent, MeOH/H_2_O 97/3, v/v (10 mmol/L CH_3_COONH_4_, 0.1% acetic acid)), were flowed by the pump A and the pump B, respectively. Sample clean-up on SPE column and rinsing step is shown in Figure 1(a). At the beginning of chromatography, 50 *μ*L sample was injected into the system. After injecting the samples, the flow rate of eluent A was 4500 *μ*L/min and the flow rate of eluent B was 400 *μ*L/min for the first 0.8 min. The flow of eluent A enriched the sample on SPE column, and the flow of eluent B entered and equilibrated analytical column in this same period (Figure 1(a)). At 0.8 min after injecting of the samples, the position of valves switched from Figure 1(a) to Figure 1(b). The flow rates of both eluent A and B were changed to 300 *μ*L/min in this period. The timetable of flow rates for the two configured isocratic pumps was shown in Table 1. The position of switching valve returned back as shown in Figure 1(a) at 2.3 min. Until next injection, this position remained constant for re-equilibration.

In this combined system, fast and reliable extraction procedure was performed, and the analytes were eluted from the precolumn to the analytical column by eluent B. High organic content the eluent B (MeOH/H_2_O 97/3, 10 mmol/L CH_3_COONH_4_, 0.1% acetic acid) is compatible with TurboIonSpray source and provides reduced band extension. Multiple reaction monitoring (MRM) mode is used for analytes with similar retention times because of eluent B is not enough to separate the analytes in phenyl-hexyl column.  Table 2 shows the retention times of immunosuppressive drugs. The total run time of online SPE, LC-MS/MS detection, and equilibrium was 2.5 min for all analytes, including two internal standards.

Mass spectrometric detection with a TurboIonSpray interface was used for detection of the immunosuppressive drugs. The turbogas temperature was set at 325°C, and the ion spray voltage was adjusted to 5500 V. High-purity nitrogen was used as curtain gas (20 psi) and collision gas (8 psi). Air was used as nebulizer gas (12 psi) and drying gas. The mass spectrometer was operated at unit resolution for both quantifier and qualifier in the MRM mode, with a dwell time of 40 ms per MRM channel. API 3200 MS/MS parameters of declustering potential (DP), entrance potential (EP), collision cell entrance potential (CEP), collision energy (CE), collision cell exit potential (CXP), and the retention times were presented in Table 2 for all targeted immunosuppressive drugs and internal standards. Analyst 1.4.2 software was used for the control of equipment, data acquisition, and analysis.

## 3. Results

**Figure 2:**
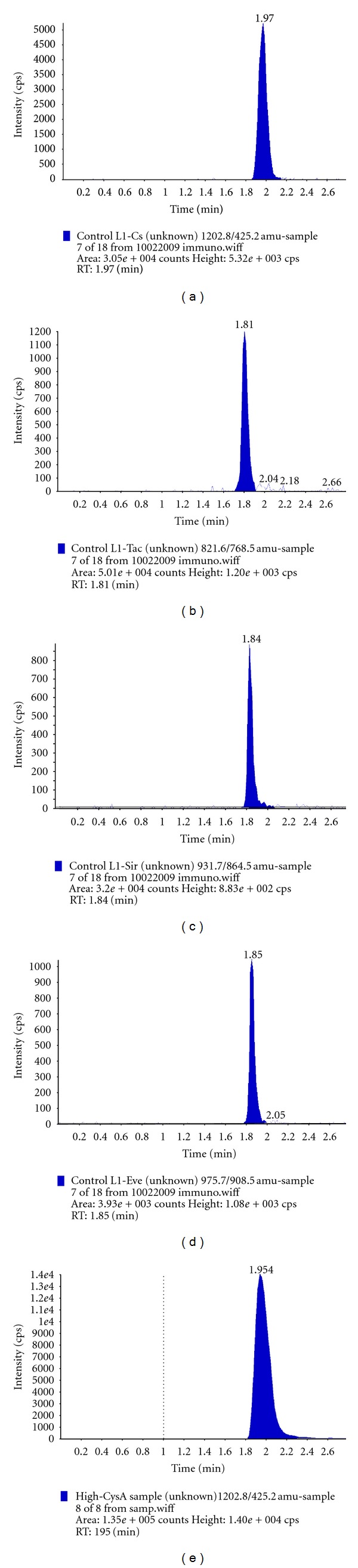
Extracted ion chromatograms of (a) cyclosporin A, (b) tacrolimus, (c) sirolimus, and (d) everolimus from whole blood control and (e) cyclosporin A from patient sample.

An LC-MS/MS assay permits robust, rapid, sensitive, selective and simultaneous quantification of four immunosuppressive drugs (CyA, TRL, SRL, and RAD) in whole-blood samples. We used online extraction of samples, a short phenyl hexyl analytic column and mobile phase containing high organic solvent, so that the total analysis time was less than 2.5 min for all analytes. The ion chromatograms of all immunosuppressive drugs extracted from both control and patient whole blood samples are shown in Figure 2.

For system performance evaluation, we determined the limits of detection (LOD), the lower limits of quantification (LOQ), the squared correlation coefficients (*R* ^2^), the recovery rates, the relative standard deviation values (R.S.D.), and the accuracy values. LOD and LOQ were estimated by injecting whole blood samples spiked with the analytes at low concentrations. The LOD was set on the basis of a 3 : 1 signal-to-noise ratio, and the LOQ was estimated on the basis of a 10 : 1 signal-to-noise ratio. The LOD was 1.4 *μ*g/L for TRL, 0.72 *μ*g/L for SRL, 1.15 *μ*g/L for RAD, and 5.6 *μ*g/L for CyA. The LOQ was 4.0 *μ*g/L for TRL, 1.8 *μ*g/L for SRL, 3.1 *μ*g/L for RAD, and 15.4 *μ*g/L for CyA. The assay was linear over the range of 4–250 *μ*g/L for TRL (*r* ^2^ = 0.9998), 1.8–250 *μ*g/L for SRL (*r* ^2^ = 0.9998), 3.1–250 *μ*g/L for RAD (*r* ^2^ = 0.9998) and 15.4–4400 *μ*g/L for CyA (*r* ^2^ = 0.9998) (Table 3).

The analytical recovery results for CyA, TRL, SRL, and RAD at low concentrations were 102.1%, 95.0%, 94.3%, and 95.9%, respectively. The analytical recovery results for high concentrations of CyA, TRL, SRL and RAD were 98.5%, 92.8%, 95.7% and 94.9%, respectively (Table 3). The commercial control whole blood samples that have three different concentrations of immunosuppressive drugs were measured 20 times in the same run for intraday precision and were measured 20 times in consecutive days for interday precision. The results of these precision measurements were presented in Table 3. Coefficient of Variation (CV) for intraday precision was below 5% for all analytes and concentrations. CV for inter-day precision did not exceed 4.5% for SRL, 4.3% for TRL, 5.9% for RAD, and 6.2% for CyA. Determination of mean accuracy of CyA, TRL, SRL, and RAD measurements was studied at commercial whole blood control samples, which was calculated to be 100.2%, 99%, 100.3% and 97.9%, respectively (Table 3).

To avoid sample matrix effects, online SPE and LC-MS/MS were used together. Phenyl-Hexyl analytical column with high organic solvent mobile phase (methanol/water 97/3, v/v) was used in this system for eliminating residual interfering effects of the blood matrix [14, 16]. Thus residual matrix interferences passed the column without retentions.

The ion chromatograms of all immunosuppressive drugs extracted from a control whole blood and a patient's whole blood for each analyte are shown in Figure 2. Because of the very short total analysis time (2.5 min), the peaks of each immunosuppressive drug in the chromatogram were not chromatographically separated from each other (Table 1). Therefore, the peaks of each immunosuppressive drug were separated with MRM mode. There were no ion suppression effects of analytes to each other.

## 4. Discussion

In this study, we used combination of online SPE and LC-MS/MS for simultaneous determination of four immunosuppressive drugs in whole blood samples of patients with organ transplantation. This method has some advantages compared to offline SPE LC-MS/MS and immunoassays. In this method, there is no overestimation of drugs concentrations due to nonspecific cross-reaction from their metabolites encountered in immunoassays [10]. The total procedure of online SPE LC-MS/MS method is very simple and requires short time unlike offline SPE LC-MS/MS. LC-MS/MS has high specificity and sensitivity for determining drugs concentrations and has been used as standard method for TDM. Additionally, the measurement of more than one drug from the same sample is possible in this chromatographic method. But usage of LC-MS/MS method for TDM in routine clinical practice may be limited due to the following factors: the first establishment of instruments can be costly; highly trained personnel are required; total analysis time from sample preparation until results are achieved is longer. Combining online SPE with LC-MS/MS method has the following advantages: minimizing the matrix effect due to fast and simple extraction procedure, noise reduction, limitation of unspecific peaks, and more samples analyzing with one analytical column. Therefore, compared to offline SPE LC-MS/MS technique, online SPE LC-MS/MS is more useful for routine TDM in clinical practice [11, 16].

We used a short phenyl-hexyl reverse phase column as analytical column and high flow rate of high organic solvent (methanol/water 97/3, v/v) as a mobile phase, so that the elution time of all immunosuppressive drugs was shorter than 2 min. Because of this short analytical time, there were no retention time differences for all immunosuppressive drugs. To solve this problem, MRM detection mode of LC-MS/MS was used, which allows effective separation of chromatographic peaks.

Interfering effects of the sample matrix are an important problem in quantification of these four immunosuppressive drugs. In this study, better matrix component elimination was achieved by using online SPE in front of LC-MS/MS analysis compared to direct LC-MS/MS analysis. Additionally, the blood matrix was also eliminated with the help of a phenyl-hexyl reverse-phase column and mobile phase, which contain high organic solvent [14, 16]. Therefore, the residue of matrix passed the column without retentions.

We analyzed more than 1000 whole blood samples with one perfusion column for online SPE and one phenyl-hexyl column for LC-MS/MS analyses at 60°C. In the evaluation of analytical column performance, we observed that there were no peak broadening, peak tailing, peak splitting, increased pressure of the column, or shifting on retention times of all analytes.

As a conclusion, we have described an online SPE-LC-MS/MS method for simultaneous determinations of presently interesting four immunosuppressive drugs (CyA, TRL, SRL, and RAD) in whole blood samples. In this method the sample preparation procedure was extremely simple, the cost of total analyses was very low, and the time needed to perform these measurements was very short. To reduce time of analyses and to solve sample matrix problems, we used online SPE perfusion column combined with a short phenyl-hexyl analytical column and MRM mode of highly selective MS/MS. This method also provided low CV levels for intraday and interday precisions and specificity and sensitivity. This method permits robust, simultaneous, and low-cost analyses of TDM with multiple immunosuppressive drugs.

## Figures and Tables

**Table 1:** Timetable for pump configuration.

Time (min)	Flow rate (*μ*L/min)	
	Eluent A	Eluent B
0.00	300	400
0.05	4500	300
0.75	4500	400
0.80	4500	300
0.85	300	300
2.20	300	300
2.25	4500	300
2.45	3300	300
2.50	300	300

Eluent A: MeOH/H_2_O (50/50, v/v); eluent B: MeOH/H_2_O (97/3, v/v; 10 mmol/L CH_3_COONH_4_, 0.1% acetic acid).

**Table 2:** LC-MS/MS parameters (MRM transition, DP, EP, CEP, CE, and CXP) and retention times of four immunosuppressive drugs and internal standards.

	CyA	TRL	SRL	RAD	Ascomycin IS	CyD IS
MRM-transition	1202.80 → 425.20	821.60 → 768.50	931.66 → 864.50	975.65 → 908.50	809.59 → 756.40	1216.90 → 425.50
DP (V)	100.00	50.63	41.25	44.38	50.63	110.00
EP (V)	10	10	10	10	10	10
CEP (V)	39.85	34.88	38.52	39.97	34.49	47.93
CE (V)	68	29	23	250	29	69
CXP (V)	4	12	14	14	12	4
Retention time (min)	1.97	1.81	1.84	1.85	1.81	2.00
Retention time RSD% (min)	0.245	0.137	0.186	0.142	0.148	0.236

CyA: cyclosporin A, TRL: tacrolimus, SRL: sirolimus, RAD: everolimus, CyD: cyclosporin D, IS: internal Standard, MRM: multiple reaction monitoring, DP: declustering potential, EP: entrance potential, CEP: collision cell entrance potential, CE: collision energy, CXP: collision cell exit potential, and V: volt.

**Table 3:** Method performance parameters.

	CyA	TRL	SRL	RAD
LOD (*μ*g/L)	5.6	1.4	0.72	1.15
LOQ (*μ*g/L)	15.4	4	1.8	3.1
Linearity^a^ (*R* ^2^)	0.9998	0.9998	0.9998	0.9998
Recovery^b^ (%)				
Concentration 1	102.1	95	94.3	95.9
Concentration 2	98.5	92.8	95.7	94.9
Intraday CV (%) (LI)^c^	3.2	2.8	3.2	2.4
Interday CV (%) (LI)^c^	3.2	4.3	4.5	4.1
Intraday CV (%) (LII)^d^	3.3	2.2	2.1	1.7
Interday CV (%) (LII)^d^	4.7	4.3	4.3	5.9
Intraday CV (%) (III)^e^	4.5	3.5	4.8	3.0
Interday CV (%) L(III)^e^	6.2	3.8	3.0	3.99
Accuracy (%)	100.2	99.0	100.3	97.9

CyA: cyclosporin A, TRL: tacrolimus, SRL: sirolimus, RAD: everolimus, and LOD: limit of detection, LOQ: lover limits of quantification.

^a^ *c* = 15.4–4400 *μ*g/L (CyA), 1.8–250 *μ*g/L (SRL), 3.1–200 *μ*g/L (RAD), and 4–200 *μ*g/L (TRL).

^b^ *c*1 = 100 *μ*g/L (CyA), *c*1 = 10 *μ*g/L (SRL, RAD, TRL), *c*2 = 500 *μ*g/L (CyA), and *c*2 = 100 *μ*g/L for (SRL, RAD, and TRL).

^c^ *c* = 50 *μ*g/L (CyA), *c* = 4 *μ*g/L (TRL), *c* = 1.8 *μ*g/L (SRL), *c* = 3.1 *μ*g/L (RAD).

^d^ *c* = 100 *μ*g/L (CyA), *c* = 20 *μ*g/L (TRL), *c* = 7.2 *μ*g/L (SRL), and *c* = 12.4 *μ*g/L (RAD).

^e^ *c* = 500 *μ*g/L (CyA), *c* = 40 *μ*g/L (TRL), *c* = 18 *μ*g/L (SRL), and *c* = 31 *μ*g/L (RAD).

## Abbreviations

LC-MS/MS: Liquid chromatography-tandem mass spectrometry
CyA: Cyclosporine A
TRL: Tacrolimus
SIR: Sirolimus
RAD: Everolimus.
